# Multimodal Large Language Models for Prognostic Prediction in Cervical Cancer Treated with Definitive Chemoradiotherapy: An Exploratory Study of Systematic Multimodal Data Integration

**DOI:** 10.3390/cancers18193136

**Published:** 2026-09-28

**Authors:** Zhaoqi Gu, Chen Wang, Qizhen Zhu, Weiping Wang, Yidong Zhang, Ke Hu

**Affiliations:** Department of Radiation Oncology, Peking Union Medical College Hospital, Chinese Academy of Medical Sciences & Peking Union Medical College, Beijing 100730, China; guzhaoqi@pumch.cn (Z.G.); wangchen_onco@163.com (C.W.); zhuqizhen@pumch.cn (Q.Z.); wangweiping@pumch.cn (W.W.); zhangyidong@pumch.cn (Y.Z.)

**Keywords:** cervical cancer, large language models, multimodal artificial intelligence, prognostic prediction, MRI report generation, chemoradiotherapy, prompt engineering, LLM-as-a-judge

## Abstract

Background/Objectives: About 25–35% of patients with cervical cancer treated with definitive concurrent chemoradiotherapy (CCRT) relapse within five years, and non-imaging clinical factors stratify their risk only modestly. We evaluated whether a general-purpose multimodal large language model (MLLM), used without fine-tuning, could estimate recurrence risk in this setting. Methods: In this retrospective single-center study, 82 patients treated with definitive radiotherapy (79/82 with concurrent platinum-based chemotherapy) who had a complete pretreatment MRI report were analyzed. Gemini 3.1 Pro generated a structured report from pelvic MRI images and, separately, estimated recurrence/metastasis risk from clinical, laboratory, treatment and imaging data. Because treatment cycles and overall treatment time actually completed were included, this was a retrospective treatment-complete risk assessment rather than a strictly pretreatment prediction. Five prompting strategies differing only in their inputs—an ablation of input modalities—were each run three times; AI reports were graded against paired human reports on a 14-item rubric by an independent model (Claude Opus 4.6). Results: Forty patients (48.8%) relapsed. Discrimination rose from an AUC of 0.704 with a clinical baseline to 0.790 with the full multimodal input (95% CI 0.687–0.883; ΔAUC +0.085). This gain was significant on our primary two-sided bootstrap test after Holm correction for ten pairwise comparisons (*p* = 0.032; a DeLong sensitivity analysis supported some but not all the imaging-benefit comparisons). Adding MRI report text significantly improved the AUC over the clinical baseline, and no statistically significant difference was detected between the human-report and AI-report strategies; this was not an equivalence or non-inferiority test; and the further lymph-node increment was not statistically significant. The AI reports scored 68.8% against an LLM judge on the rubric, with apparent overcalling of parametrial invasion and an inability to assess lymph nodes within the supplied field of view; all strategies underestimated absolute risk (E/O 0.78–0.83). Conclusions: A non-fine-tuned MLLM can integrate multimodal data into a prognostic estimate, but its automatically generated MRI reports are frequently discordant with radiologist reports in specific ways and require expert review and external validation before clinical use.

## Simple Summary

About a quarter to a third of women treated with definitive chemoradiotherapy for cervical cancer eventually relapse; therefore, identifying higher-risk patients early would help individualize treatment intensity and follow-up. Multimodal large language models can read images and text together and reason over many kinds of clinical data without being trained for the specific task. In 82 patients, we assessed whether one such general-purpose model (Gemini 3.1 Pro) could both generate a structured report from pelvic MRI images and combine clinical, laboratory, treatment and imaging data to estimate recurrence risk. Supplying an MRI report improved the prediction, and the best setup reached an AUC of approximately 0.79, though this specific gain over clinical data alone held on our primary statistical test but not on a secondary sensitivity check. When we checked the model’s own MRI reports against the radiologists’ reports, however, they were often discordant in specific and clinically important ways—overcalling parametrial invasion relative to the radiologist’s report and being unable to assess lymph nodes. Therefore, they are not yet safe to use without oversight. We report the pipeline and its baseline results for others to build upon.

## 1. Introduction

Cervical cancer remains one of the most common gynecologic cancers, with an estimated 604,000 new cases and 342,000 deaths in 2020 and a similar burden in the 2022 global figures [1,2]. For women treated with definitive CCRT—external-beam radiotherapy (EBRT) with intracavitary brachytherapy and platinum-based concurrent chemotherapy—five-year disease-free survival is still only about 60–70%, despite steady improvements in delivery (IMRT, VMAT and helical tomotherapy), and roughly a quarter to a third experience local or distant relapse within five years [3,4,5,6].

Reliable stratification before treatment would help, but the established clinical markers—FIGO stage, tumor size, nodal status, hemoglobin and SCC-Ag [7,8,9,10,11,12]—each carry only limited discriminatory weight on their own. Multimodal deep-learning models such as CerviPro (n = 1018), which incorporate clinical and localization-CT data, reached a C-index of 0.81 internally but only 0.66–0.70 externally [13]. MRI, and particularly its T2-weighted and diffusion-weighted sequences, is central to local staging, parametrial assessment and response evaluation [14,15,16]. Radiomic models built on MRI have also reported a concordance index of 0.65 on external validation [17] or an AUC of 0.70–0.80 in single centers [18], though they depend on expert segmentation and center-specific pipelines [19].

Large language models take a different route. Frontier systems such as GPT-4 and Gemini answer oncology questions and medical examinations competently without task-specific training [20,21,22,23,24], and vision-language models can describe medical images to some extent [25,26]. An early study of LLM-based prognostication in laryngeal and hypopharyngeal cancer reported an AUC near 0.73 [27]. Set against this, LLMs still lag behind conventional machine learning on structured tabular prediction (AUC ~0.60–0.63 versus 0.85–0.89) and are strongest where the input is rich free text [28]. They also fabricate findings and show confirmation bias, which is a real concern once images are involved [29,30,31].

What has not been tested is whether a pretrained MLLM can, without fine-tuning, (i) turn selected tumor-centered images from pelvic MRI sequences into a clinically structured report of acceptable quality and safety, and (ii) fold that report, together with the rest of the clinical record, into a prognostic estimate. We addressed both in 82 patients treated with definitive CCRT, using five prompts (each run three times) in an ablation of input modalities to weigh the contribution of each data modality and a rubric-based audit to characterize the AI reports. 

## 2. Materials and Methods

### 2.1. Study Design and Population

We retrospectively identified consecutive patients with histologically confirmed cervical cancer who received definitive radiotherapy (EBRT with intracavitary brachytherapy), usually with concurrent platinum-based chemotherapy, at Peking Union Medical College Hospital between November 2011 and December 2016. Patients were eligible if a pretreatment pelvic MRI with a complete radiologist report was available; those with incomplete treatment or follow-up records, or without a human MRI report, were excluded. Of 105 patients initially identified, 23 were excluded (13 without a complete pretreatment radiologist MRI report, 3 with incomplete treatment records and 7 with incomplete follow-up records), leaving 82 who met these criteria (median follow-up 5.0 years, range 0.4–10.7); three of them received radiotherapy without concurrent chemotherapy (Supplementary Figure S1). The outcome was any local or regional recurrence or distant metastasis during follow-up (label 1) versus no relapse (label 0). The study was approved by the Institutional Review Board of Peking Union Medical College Hospital (approval no. I-26PJ2020; project no. K11323; approved 24 August 2026); informed consent was waived given the retrospective use of de-identified data.

### 2.2. Clinical and Treatment Data

For each patient, we recorded 59 variables: demographics and pathology (age, histology, grade, lymphovascular space invasion, growth pattern); examination (palpable tumor size, parametrial and vaginal involvement); FIGO stage, 2009 and 2018 [9]; pretreatment laboratory values (white-cell, neutrophil and lymphocyte counts, hemoglobin, platelets, ALT, creatinine, SCC-Ag, CA125); imaging (chest and abdominal CT, PET/CT, and the structured MRI report); and treatment (EBRT technique and dose, nodal and parametrial boosts, number of brachytherapy fractions, chemotherapy regimen and cycles, and overall treatment time).

### 2.3. MRI Acquisition and Image Preparation

Pretreatment MRI was performed at 3.0 T (Discovery MR750, GE Healthcare, Waukesha, WI, USA) with a standard protocol: axial T1; sagittal and axial T2; contrast-enhanced fat-suppressed T1 (LAVA-Flex) in three planes; and DWI at b = 0 and 800 s/mm^2^ [14]. For each sequence, we cropped the slice showing the largest tumor cross-section and exported it as a JPEG. We chose tumor-centered cropping to preserve lesion visibility and spatial resolution within the model’s image-input budget; whole-image resizing and sliding-window or multi-tile processing were not evaluated. This necessarily narrowed the field of view, so the pelvic and para-aortic nodal stations were usually out of frame. This is an input limitation, not evidence of intrinsic inability to assess lymph nodes.

### 2.4. Module 1: VLM-Based AI MRI Report Generation

The same underlying model, Gemini 3.1 Pro (gemini-3.1-pro, via API), is referred to as a vision-language model (VLM) in this section because Module 1 takes images as input; the identical model used in text-only mode for Module 2 (Section 2.6) is referred to as an LLM/MLLM, consistent with the rest of the manuscript. The system prompt cast the model as a gynecologic-oncology radiologist and required a four-part report: (i) tumor site, shape and size; (ii) T1, T2 and DWI signal and enhancement; (iii) local extension (parametrium, vagina, corpus, bladder/rectum); and (iv) lymph nodes. To discourage fabrication, the prompt asked for explicit size criteria to separate tumor from edema or fibroid, mandatory DWI reporting, and non-committal nodal wording. The selected cropped slice from each sequence was sent as a base64-encoded JPEG; images were de-identified and DICOM metadata removed before transmission.

### 2.5. Quality Assessment of AI MRI Reports

#### 2.5.1. Clinical Rubric

The 14-item rubric was developed by the study team before scoring began, based on the structured reporting elements required of Module 1 and standard elements of cervical-cancer MRI staging reports. Taking the human report as reference, we scored each AI report on 14 items in four domains: tumor description (location a, morphology b, maximum craniocaudal diameter c, maximum axial size d); signal (T1/T2 e, DWI f, enhancement g); local extension (stromal ring h, parametrium i, junctional zone j, fornix/corpus k, lower vaginal third l, rectum/bladder m); and lymph nodes (n). Size items used a sliding scale (error ≤5 mm, 1.0; ≤10 mm, 0.8; ≤15 mm, 0.5; >15 mm, 0), comparing like axis and using the larger error. Items not mentioned in the reference were marked N/A and dropped from the denominator; therefore, each report score is the fraction of applicable points earned (Table S1; see Appendix A).

#### 2.5.2. LLM-As-a-Judge Evaluation

Claude Opus 4.6 (Anthropic, San Francisco, CA, USA) applied the rubric to all 82 report pairs. We deliberately chose a model from a different family to grade the Gemini output, ensuring that the judge and candidate shared no weights [32]; the 82 cases were scored in nine batches (each report scored once, rather than being replicated). We also computed BLEU and ROUGE and examined how well they tracked the rubric, since such n-gram metrics are known to correlate poorly with the clinical accuracy of generated reports [33].

#### 2.5.3. Independent Expert Validation

To check whether the LLM-as-a-judge rubric agrees with human radiologists, we sampled 18 of the 82 report pairs (about 22%, stratified by event type, fixed seed) and had two senior radiologists independently score the same 14-item rubric on the paired reference and AI-generated report for each case, blinded to Claude’s prior scores and rationale, the patient’s outcome, and the model’s predicted risk score. After both completed independent scoring, they discussed the items on which they disagreed and reached a consensus. This validates whether the scoring method used to grade AI-generated reports against reference reports agrees with independent human raters performing the same text-comparison task—it does not establish ground truth against the underlying MRI images, which would require an independent expert re-read. Absolute-agreement ICC (two-way random effects, single measures) and weighted kappa (linear score-distance weights) were used for reliability; the full protocol is set out in Supplementary Methods S6.

### 2.6. Module 2: LLM-Based Prognostic Prediction

#### 2.6.1. Overall Framework

For prognostication, Gemini 3.1 Pro was queried in text only [23]. Each patient prompt asked for a structured reasoning across six domains, a recurrence/metastasis probability (0–100%), the ten most influential risk factors, and the likely pattern and timing of relapse. The probability served as the continuous score for the AUC; temperature was fixed at 0.7.

#### 2.6.2. Systematic Prompting-Strategy Design

The five prompts differed only in the data provided, forming an ablation of input modalities such that the change in performance between them measured the value of the added data. Each was run three times per patient (15 prediction sets) to gauge the run-to-run variation at temperature 0.7:

Strategy A: clinical data + laboratory indices + full treatment information (EBRT technique, dose, brachytherapy scheme, chemotherapy cycles, etc.).

Strategy B: clinical data + laboratory indices + simplified treatment information (labeled only as “definitive concurrent chemoradiotherapy”, without specific parameters).

Strategy C: clinical data + laboratory indices + treatment information + human MRI report.

Strategy D: clinical data + laboratory indices + treatment information + AI-generated MRI report.

Strategy E: as strategy D, but with the AI-generated report’s own lymph-node section text replaced with a human lymph-node description (hybrid strategy).

Because the strategies form an ablation of input modalities, each pairwise contrast isolates one data source: A versus B isolates the effect of treatment-detail granularity, A versus C (or D) the incremental prognostic value of the MRI report, C versus D the human-versus-AI report comparison, and D versus E the effect of replacing the AI-generated lymph-node text with a human lymph-node description. The difference in AUC (ΔAUC) between paired strategies is our measure of incremental value. For strategies C, D and E, an independent audit found that two of the three originally saved runs per strategy had drifted from the intended treatment or MRI-report fields; those two runs were re-executed with inputs re-verified on a field-by-field basis against the clinical record (labeled “-rerun” in the corresponding results tables), and only these verified-input runs are used throughout this manuscript. The full prompts are provided in the Supplementary Materials.

### 2.7. Statistical Analysis

The primary measure was the AUC for the binary outcome. For each strategy, we averaged the three runs’ predicted probabilities and computed the AUC of that mean, with 95% confidence intervals from 5000 patient-level bootstrap resamples (percentile method, seed 42). We also report the range and SD of the three single-run AUCs. For the ten pairwise strategy comparisons, the primary significance test was the two-sided bootstrap tail probability from the same resampling scheme, with the Holm procedure as the primary correction for multiplicity across the ten comparisons and the Benjamini–Hochberg procedure reported alongside. The DeLong method for correlated ROC curves [34] was run as a sensitivity analysis on the same ten comparisons, with its own Holm correction, to check whether conclusions were sensitive to the choice of test. Both sets of results are reported rather than only the more favorable one. Classification performance (precision, recall, specificity, accuracy, F1) was estimated by patient-level repeated stratified five-fold cross-validation: the F1-optimal threshold was selected on the four training folds only and applied to the held-out fold, so threshold selection and evaluation never used the same patients; 95% confidence intervals were obtained by wrapping this entire procedure in an outer patient-level bootstrap (500 resamples). Calibration was summarized by the Brier score, Brier skill score, E/O ratio, and calibration-in-the-large (the intercept of a logistic recalibration of the outcome on the predicted log-odds with the slope fixed at one [35]). Because the original prompt asked for an unanchored recurrence/metastasis probability rather than a probability at a specified time horizon, we treat the model output as an uncalibrated risk score rather than a validated absolute risk, and separately report a sensitivity analysis restricted to the subset of patients with a determinate five-year status (event before five years, or event-free with at least five years of follow-up; 4 of 42 event-free patients with under five years of follow-up were excluded from this subset only). We then compared local recurrence and distant metastasis, each against the event-free group, using the same strategies and methods as the primary analysis; these subgroup comparisons are descriptive and were not corrected for multiple testing. Significance on the primary analysis was set at *p* < 0.05 after Holm correction (* *p* < 0.05, ** *p* < 0.01, both after correction). Analyses used Python 3.13 with NumPy 2.3.5, SciPy 1.16.3 and scikit-learn 1.8.0; the final inputs were checked before analysis, and the strategy configurations, run parameters and random seeds are recorded for reproducibility.

## 3. Results

### 3.1. Patient Characteristics

The 82 patients had a median age of 51.5 years (range 25–72). Squamous cell carcinoma predominated (75, 91.5%), with four adenocarcinomas (4.9%) and three adenosquamous carcinomas (3.7%). By FIGO 2018, IIB (37, 45.1%) and IIIC1(r) (18, 22.0%) were the largest groups, followed by IB (10, 12.2%), IIA (7, 8.5%), IIIC2(r) (5, 6.1%) and IIIB (5, 6.1%); by FIGO 2009, most were stage IIB (51, 62.2%), with 11 (13.4%) IIIA/IIIB, 12 (14.6%) IB and 8 (9.8%) IIA (Table 1).

All patients received EBRT (IMRT 63.4%, VMAT 28.0%, TOMO 8.5%) with intracavitary brachytherapy (mostly 30 Gy/5 fractions, 76.8%); 79 of 82 (96.3%) also received concurrent platinum-based chemotherapy (median five cycles, range 0–6), with the remaining three receiving radiotherapy alone—one because of pre-existing myelofibrosis and two because of renal insufficiency. The EBRT prescription was usually 50.4 Gy/28 fractions (92.7%). The median overall treatment time was 52 days (range 43–95), median pretreatment SCC-Ag 6.3 ng/mL (range 0.3–55.8) and median hemoglobin 130 g/L (range 80–155). The sample size is the full retrospective cohort meeting the eligibility criteria, and we rely on 95% confidence intervals throughout to convey the precision of each estimate rather than a prespecified target power. Over a median follow-up of 5.0 years, 40 patients (48.8%) relapsed—15 local, 23 distant and two both—while 42 (51.2%) remained event-free.

**Table 1:** Baseline characteristics of the cohort (n = 82).

Characteristic	Category	n (%) or Median [Range]
Age (years)	Median [range]	51.5 [25–72]
Histology	Squamous cell carcinoma	75 (91.5%)
	Adenocarcinoma	4 (4.9%)
	Adenosquamous carcinoma	3 (3.7%)
Differentiation	Well	8 (9.8%)
	Moderate	24 (29.3%)
	Poor	14 (17.1%)
	Not recorded	36 (43.9%)
FIGO 2009 stage	IB	12 (14.6%)
	IIA	8 (9.8%)
	IIB	51 (62.2%)
	IIIA/IIIB	11 (13.4%)
FIGO 2018 stage	IB1–IB2	10 (12.2%)
	IIA	7 (8.5%)
	IIB	37 (45.1%)
	IIIB	5 (6.1%)
	IIIC1(r)	18 (22.0%)
	IIIC2(r)	5 (6.1%)
EBRT technique	IMRT	52 (63.4%)
	VMAT	23 (28.0%)
	TOMO	7 (8.5%)
EBRT dose	50.4 Gy/28 fx	76 (92.7%)
Brachytherapy	30 Gy/5 fx	63 (76.8%)
Concurrent chemotherapy	Cisplatin-based	79 (96.3%)
Chemotherapy cycles	Median [range]	5 [0–6]
Treatment time (days)	Median [range]	52 [43–95]
Pretreatment SCC-Ag (ng/mL)	Median [range]	6.3 [0.3–55.8]
Pretreatment hemoglobin (g/L)	Median [range]	130 [80–155]
Outcome	Recurrence/metastasis (label = 1)	40 (48.8%)
	Local recurrence	15 (18.3%)
	Distant metastasis	23 (28.0%)
	Local + distant	2 (2.4%)
	Event-free (label = 0)	42 (51.2%)
Median follow-up (years)	Median [range]	5.0 [0.4–10.7]

### 3.2. Module 1: Quality of AI-Generated MRI Reports

Gemini produced a complete four-section report for every case. Graded against the human reports, the rubric gave a mean patient-level normalized rubric score (each case’s summed item scores divided by its own count of applicable items, then averaged over 82 cases) of 68.8%. This is a different, and slightly higher, statistic than the mean of the 14 item-level column averages (68.1%), which is what Figure 1A plots—though the score varied sharply by domain (Table 2a,b; Figure 1): 18 reports (22.0%) scored 80–100%, 42 (51.2%) scored 60–80%, 21 (25.6%) scored 40–60%, and one (1.2%) scored below 40%.

**Table 2:** (**a**) Rubric scores of AI MRI reports by dimension (n = 82). (**b**) Rubric scores of AI MRI reports for the 14 items (n = 82).

(**a**)				
**Dimension**	**Included Items**		**Weighted Mean**	**Rating**
Tumor description (a–d)	Location, morphology, vertical diameter, cross-sectional size		0.67	Moderate
Signal characteristics (e–g)	T1/T2, DWI, enhancement		0.83	Excellent
Local invasion (h–m)	Stroma, parametrium, junctional zone, fornix, vagina, rectum/bladder		0.65	Moderate
Lymph node (n)	Lymph-node assessment		0.60	Moderate
(**b**)				
**Item**	**Applicable n**	**Mean**	**Full-Score Rate**	**Zero-Score Rate**
a. Location	82	0.84	70%	1%
b. Morphology	65	0.58	34%	17%
c. Vertical diameter	60	0.59	28%	32%
d. Cross-sectional size	47	0.61	30%	28%
e. T1/T2	73	0.89	89%	11%
f. DWI	56	0.91	91%	9%
g. Enhancement	64	0.68	67%	31%
h. Stromal-ring continuity	64	0.86	84%	12%
i. Parametrial invasion	63	0.36	33%	62%
j. Junctional zone	50	0.38	38%	62%
k. Fornix/uterine corpus	75	0.50	20%	20%
l. Lower one-third of vagina	72	0.96	96%	4%
m. Rectum/bladder	78	0.77	77%	23%
n. Lymph node	81	0.60	49%	30%

Scoring was performed by Claude Opus 4.6, using an LLM-as-a-judge approach, providing cross-model independent evaluation. Full-score rate = proportion scoring 1.0; zero-score rate = proportion scoring 0. “Applicable n” is the number of cases in which the reference report described the item (N/A excluded).

Signal description was the strongest area of agreement—DWI and T1/T2 matched the reference report in 91% and 89% of cases, and the lower vaginal third in 96%. Size was the first area of discordance: craniocaudal diameter and axial size were frequently larger in the AI report than in the reference (means 0.59–0.61; zero-score rate 28–32%), particularly for tumors under 2 cm. The most clinically important discordances were in local extension. In the cases where the reference report recorded no parametrial invasion, the AI report stated parametrial invasion (typically ‘bilateral strip-like parametrial infiltration’) in most of them (item i: mean 0.36, zero-score rate 62%), and showed a comparable rate of discordance for the junctional zone (item j: 62%)—both are rubric-defined discordance rates against the reference report, not an independently confirmed imaging-level false-positive rate. Of these two, only parametrial invasion is itself a FIGO 2018 staging criterion; junctional-zone or corpus extension does not by itself raise the reported stage, so we no longer group the two together as equally stage-changing. Rectum and bladder were generally concordant with the reference (full-score rate 77%) but under-reported when the reference suggested invasion. Lymph nodes showed a distinct, structurally explainable pattern of discordance (zero-score rate 30%): when the reference described a node with a short axis over 1 cm, the AI report typically did not, because the cropped images did not contain the nodal stations. BLEU and ROUGE tracked none of this (all |r| < 0.2), which is why we relied on the rubric rather than word-overlap scores [33].

To check whether these LLM-as-a-judge scores agree with human raters, two senior radiologists independently scored the same rubric on an 18-case stratified subset (Section 2.5.3, Table 3). The two radiologists’ independent scores agreed with each other at a level conventionally read as moderate-to-good (absolute-agreement ICC 0.743), though the 95% CI (0.299–0.918) is wide at this sample size and should not be read as a precise estimate. Claude’s scores were systematically lower than the human consensus reached after the two radiologists discussed and resolved their disagreements (mean normalized score 0.648 for Claude, using item scores recomputed directly from the per-item rubric data versus 0.807 for the human consensus; ICC 0.283, 95% CI 0.077–0.496, conventionally read as poor agreement). Item-level agreement between Claude and the human consensus was higher for parametrial invasion (weighted kappa 0.90) than for morphology, cross-sectional size, tumor location and enhancement pattern (weighted kappa 0.10–0.38), though the parametrial-invasion comparison itself rests on only 10 of the 18 cases where both Claude and the human consensus judged the item applicable, so this should be read as consistent with, not as an independent confirmation of, the parametrial-invasion finding discussed below—it does not validate the finding across the full 82-case cohort or establish ground truth against the underlying images. Taken together, these results indicate that Claude’s rubric scoring is more consistently stringent than two independent radiologists on several items. Therefore, the 68.8% macro-average score above is best treated as a figure specific to the Claude-as-judge standard, not an independently confirmed absolute quality score.

**Table 3:** Independent radiologist validation of the LLM-as-a-judge rubric (n = 18 stratified subset). ICC(A,1) = absolute-agreement intraclass correlation, two-way random effects, single measures, with patient-level bootstrap 95% CI (3000 resamples, seed 42). Scores are the case-level normalized rubric score (sum of applicable item scores/number of applicable items). The human-consensus column reports the value agreed upon by each rater after independently scoring and then discussing the items on which they disagreed (Section 2.5.3); the Radiologist-1-vs-2 row uses their original independent scores only.

Comparison	ICC(A,1) [95% CI]	Mean Score (Row)	Mean Score (Column)	Mean |Difference|
Radiologist 1 vs. Radiologist 2 (independent)	0.743 [0.299, 0.918]	0.826	0.823	0.044
Claude vs. Radiologist 1 (independent)	0.216 [0.029, 0.441]	0.648	0.826	0.178
Claude vs. Radiologist 2 (independent)	0.247 [0.039, 0.465]	0.648	0.823	0.175
Claude vs. human consensus (post-adjudication)	0.283 [0.077, 0.496]	0.648	0.807	0.159

### 3.3. Module 2: Prognostic Prediction—Discrimination

Discrimination for the five strategies is given in Table 4 and Figure 2. For each strategy, we report the AUC of the mean predicted score over three runs, together with the individual run AUCs.

Clinical data alone gave an AUC of 0.704 (strategy A; runs 0.684–0.717, mean 0.698 ± 0.017) and 0.707 (strategy B; runs 0.700–0.705, mean 0.702 ± 0.002). The two were statistically indistinguishable (ΔAUC +0.003, Holm-corrected *p* = 1.00), so no statistically significant difference was detected between the terse and detailed treatment descriptions; strategy A was also more variable between runs than strategy B (SD 0.017 versus 0.002).

Adding the human MRI report raised the AUC to 0.784 (strategy C; three verified-input runs 0.777, 0.788 and 0.771, mean 0.779 ± 0.009); this was a gain over strategy A, which remained significant on our primary bootstrap test after Holm correction (ΔAUC +0.079, Holm *p* = 0.032) but was not confirmed by the DeLong sensitivity analysis after the same correction (Holm *p* = 0.140).

The AI report gave an AUC of 0.762 (strategy D; three verified-input runs: 0.740, 0.768 and 0.754; mean: 0.754 ± 0.014); its improvement over the clinical baseline did not reach significance after correction (ΔAUC +0.058, Holm *p* = 0.391), and it did not differ significantly from the human report (strategy C versus D: ΔAUC −0.022, Holm *p* = 1.00). This study was not designed or powered as a non-inferiority trial, so the correct reading is that no statistically significant difference was detected between the AI and human report strategies in this exploratory cohort, not that the two are equivalent. Despite its lower rubric scores, the AI report carried numerically similar prognostic information.

The full input reached 0.790 (strategy E; three verified-input runs: 0.786, 0.780 and 0.769; mean: 0.778 ± 0.009—with a relatively narrow spread), the best of the five and significantly above the clinical baselines A and B on the primary bootstrap test after Holm correction (ΔAUC +0.085 and +0.082, both Holm *p* = 0.032), though the DeLong sensitivity analysis did not confirm significance after the same correction (Holm *p* = 0.125 and 0.094). The increment over the AI-report strategy D (ΔAUC +0.027) and the difference from the human-report strategy C (ΔAUC +0.006) were not statistically significant (Holm *p* = 0.391 and 1.00).

**Table 4:** Discrimination of the prompting strategies (n = 82).

Strategy	Input Configuration	3-Run AUC	Mean ± SD	Mean-Score AUC	95% CI
A (v1–v3)	Clinical + full treatment	0.684, 0.693, 0.717	0.698 ± 0.017	0.704	[0.585, 0.816]
B (v4–v6)	Clinical + simplified treatment	0.701, 0.705, 0.700	0.702 ± 0.002	0.707	[0.590, 0.815]
C (v7, v9-rerun, v10-rerun)	Clinical + human MRI	0.777, 0.788, 0.771	0.779 ± 0.009	0.784	[0.678, 0.878]
D (v12, v16-rerun, v17-rerun)	Clinical + AI MRI	0.740, 0.768, 0.754	0.754 ± 0.014	0.762	[0.653, 0.861]
E (v14, v15-rerun × 2)	Clinical + AI MRI + human LN	0.786, 0.780, 0.769	0.778 ± 0.009	0.790	[0.687, 0.883]

AUC = area under the ROC curve; CI = bootstrap 95% confidence interval (5000 resamples); LN = lymph-node description. “Mean-score AUC” is computed from the mean of the predicted probabilities over three runs; “Mean ± SD” summarizes the three independent-run AUCs.

### 3.4. Pairwise Statistical Comparisons

On two-sided bootstrap testing with Holm correction for the ten pairwise comparisons (Table 5, Figure 3), adding a human MRI report significantly improved discrimination (A versus C, Holm *p* = 0.032; B versus C, Holm *p* = 0.045), as did the full multimodal input over the clinical baselines (A versus E and B versus E, both Holm *p* = 0.032). None of these four comparisons remained significant on the DeLong sensitivity analysis after the same correction (Holm *p* = 0.09–0.14), so we treat the imaging-benefit finding as significant on our primary test but not confirmed by a correlated-ROC sensitivity check, rather than as robust under every reasonable test. The AI-report strategy showed a non-significant increase in AUC over the baselines (A versus D, Holm *p* = 0.391; B versus D, Holm *p* = 0.391); the human lymph-node description added a non-significant increment over the AI report (D versus E, +0.027, Holm *p* = 0.391); and neither the human-versus-AI report comparison (C versus D, Holm *p* = 1.00) nor the treatment-detail comparison (A versus B, Holm *p* = 1.00) reached significance. Uncorrected *p*-values, Benjamini–Hochberg-corrected *p*-values and the DeLong statistics are all given in Table 5.

**Table 5:** Pairwise AUC comparisons between strategies (n = 82).

Comparison	ΔAUC	Bootstrap 95% CI	Bootstrap *p* (Uncorrected)	Bootstrap *p* (Holm)	Bootstrap *p* (BH)	DeLong *p* (Holm)	Sig. (Holm, Bootstrap)
A vs. C (clinical → human MRI)	+0.079	[+0.019, +0.152]	0.0036	0.032	0.013	0.140	*
A vs. D (clinical → AI MRI)	+0.058	[−0.010, +0.134]	0.098	0.391	0.139	0.477	ns
A vs. E (clinical → best multimodal)	+0.085	[+0.024, +0.160]	0.0040	0.032	0.013	0.125	*
B vs. C (simplified → human MRI)	+0.076	[+0.022, +0.139]	0.0064	0.045	0.016	0.094	*
B vs. D (simplified → AI MRI)	+0.055	[−0.003, +0.123]	0.065	0.391	0.130	0.477	ns
B vs. E (simplified → best multimodal)	+0.082	[+0.023, +0.152]	0.0032	0.032	0.013	0.094	*
A vs. B (full vs. simplified treatment)	+0.003	[−0.067, +0.083]	0.945	1.00	0.945	1.00	ns
C vs. D (human vs. AI report)	−0.022	[−0.068, +0.024]	0.351	1.00	0.439	0.990	ns
D vs. E (AI report → AI + human LN)	+0.027	[−0.003, +0.061]	0.078	0.391	0.130	0.477	ns
C vs. E (human MRI → hybrid)	+0.006	[−0.025, +0.038]	0.715	1.00	0.794	1.00	ns

Bootstrap: 5000 resamples, seed = 42, independent random-number generator per comparison; *p*-values are two-sided. * *p* < 0.05; ns, *p* ≥ 0.05. ΔAUC is based on the mean predicted score over three runs.

### 3.5. Classification Performance (Best-F1 Threshold)

Classification performance was estimated by patient-level repeated stratified five-fold cross-validation, with the decision threshold selected only on the training folds of each split and evaluated on the held-out fold; the outer 95% CIs additionally resample patients with replacement and keep every resampled copy of the same patient in the same fold (grouped cross-validation), so that no patient can contribute to both the training and the test side of a split (Table 6, Figure 4). Strategy E gave the best F1 (0.742, 95% CI 0.599–0.829); strategy D gave the best specificity (0.553, 95% CI 0.297–0.741); and strategies C and E tied for the best recall (0.885). All strategies had wide confidence intervals, reflecting the sample size. The clinical-only strategies (A, B) had the lowest specificity (0.42 and 0.29) and the most false positives; adding imaging (C, D, E) improved specificity to 0.49–0.55 without materially reducing recall, which remained high throughout (0.81–0.89 across all five strategies).

**Table 6:** Classification performance at the best-F1 threshold (n = 82).

Strategy	Threshold Method	F1 [95% CI]	Precision [95% CI]	Recall [95% CI]	Specificity [95% CI]	Accuracy [95% CI]	TP/FP/FN/TN (Mean of CV Repeats)
A (clinical + full treatment)	Grouped, repeated stratified 5-fold CV (train-fold selected)	0.669 [0.521, 0.781]	0.570 [0.419, 0.724]	0.810 [0.608, 0.918]	0.419 [0.068, 0.736]	0.610 [0.443, 0.738]	32.4/24.4/7.6/17.6
B (clinical + simplified treatment)	Grouped, repeated stratified 5-fold CV (train-fold selected)	0.643 [0.534, 0.763]	0.526 [0.419, 0.720]	0.828 [0.623, 0.949]	0.288 [0.095, 0.763]	0.551 [0.461, 0.744]	33.1/29.9/6.9/12.1
C (clinical + human MRI)	Grouped, repeated stratified 5-fold CV (train-fold selected)	0.730 [0.596, 0.828]	0.621 [0.508, 0.772]	0.885 [0.669, 0.958]	0.487 [0.373, 0.741]	0.681 [0.583, 0.795]	35.4/21.5/4.6/20.5
D (clinical + AI MRI)	Grouped, repeated stratified 5-fold CV (train-fold selected)	0.719 [0.583, 0.825]	0.638 [0.501, 0.774]	0.825 [0.661, 0.929]	0.553 [0.297, 0.741]	0.686 [0.549, 0.800]	33.0/18.8/7.0/23.2
E (clinical + AI MRI + human LN)	Grouped, repeated stratified 5-fold CV (train-fold selected)	0.742 [0.599, 0.829]	0.640 [0.512, 0.771]	0.885 [0.655, 0.950]	0.526 [0.300, 0.797]	0.701 [0.573, 0.793]	35.4/19.9/4.6/22.1

The threshold was selected only on the training folds of a patient-level repeated stratified five-fold cross-validation and evaluated on the held-out fold each time; 95% CIs additionally resample patients with replacement in an outer bootstrap that keeps every copy of a resampled patient in the same fold, so no patient contributes to both sides of a split. TP, true positive; FP, false positive; FN, false negative; TN, true negative; the TP/FP/FN/TN column reports the mean across cross-validation repeats rather than integer counts from a single fixed split, and is therefore reported to one decimal place. The results are based on the mean predicted score across three runs.

### 3.6. Event-Type Subgroup Analysis

Of the 40 events, 15 (37.5%) were local recurrence, 23 (57.5%) were distant metastasis and 2 (5.0%) both; with only two combined cases, we compared local recurrence and distant metastasis separately against the 42 event-free patients (Table 7, Figure 5).

AUCs were numerically higher for distant metastasis than for local recurrence across strategies. For distant disease, strategy E reached 0.810 (95% CI 0.692–0.909) against 0.745 for strategy A, and all imaging strategies exceeded 0.79; for local recurrence, strategies C and E were around 0.75–0.76, against only 0.624 for strategy A. The imaging gain (A→C) was numerically larger for local recurrence (+0.129) than for distant metastasis (+0.054) in this descriptive comparison; we do not infer from this alone that imaging carries more local-recurrence signal, since no formal subgroup-by-strategy interaction test was performed and the local-recurrence subgroup has only 15 events. Distant-relapse patients may also already differ from event-free ones on clinical grounds [36]. These subgroup comparisons are descriptive and were not corrected for multiple testing; a formal interaction test would be needed to support a differential-effect claim.

**Table 7:** Event-type subgroup AUC analysis (n = 82).

Strategy	Local Recurrence AUC (15 vs. 42)	95% CI	Distant Metastasis AUC (23 vs. 42)	95% CI
A (clinical + full treatment)	0.624	[0.448, 0.792]	0.745	[0.610, 0.867]
B (clinical + simplified treatment)	0.671	[0.508, 0.820]	0.725	[0.588, 0.857]
C (clinical + human MRI)	0.752	[0.604, 0.885]	0.799	[0.679, 0.901]
D (clinical + AI MRI)	0.700	[0.535, 0.848]	0.799	[0.682, 0.899]
E (clinical + AI MRI + human LN)	0.757	[0.610, 0.886]	0.810	[0.692, 0.909]
MRI increment (A → C)	+0.129		+0.054	

The combined local + distant subgroup (n = 2) was excluded owing to small size. Bootstrap: 5000 resamples, seed = 42. MRI increment = AUC for Strategy C – AUC for Strategy A.

### 3.7. Calibration Analysis

Every strategy underestimated absolute risk on point estimate, with E/O ratios of 0.78–0.83 (mean predicted score 78–83% of the observed event rate) and a positive calibration-in-the-large point estimate for all five strategies (range +0.40 to +0.54; Table 8, Figure 4); the 95% CI excluded zero for strategies A and D only, and included zero for B, C and E. Therefore, we do not claim that every strategy is significantly miscalibrated in this sample, only that the point estimates are consistently in the same direction. The reliability diagram (Figure 4E) shows the same pattern qualitatively: observed event rates exceeded the mean predicted score in most of the lower- and mid-risk quartile bins for most strategies. However, with only n ≈ 20 patients per bin, the bin-level estimates themselves carry wide uncertainty and should be read as descriptive rather than as a precise calibration function. In any case, because the original prompt asked for an unanchored probability rather than a probability at a defined time horizon, we treat the raw model output as an uncalibrated risk score rather than a validated probability, independent of this test’s significance. Strategy C had the lowest Brier score (0.203) and the highest BSS (0.188), essentially tied with strategy E (Brier 0.204, BSS 0.184). Because the original prompt did not specify a time horizon, we additionally computed discrimination on the subset of 78 patients with a determinate five-year status (all 40 events occurred within five years by the recorded time-to-event; four event-free patients with under five years of follow-up were excluded from this subset only): AUCs were materially unchanged from the full cohort (e.g., strategy E 0.793 [0.684, 0.885] versus 0.790 for the full cohort), which is reassuring but does not establish that the original, unanchored probability was calibrated to any specific time point.

**Table 8:** Calibration metrics of the strategies (n = 82).

Strategy	Input Configuration	Brier Score	BSS	E/O Ratio	Calib.-in-the-Large [95% CI]
Naive reference	BS = 0.250				
A (v1–v3)	Clinical + full treatment	0.234	0.062	0.780	+0.54 [+0.00, +1.04]
B (v4–v6)	Clinical + simplified treatment	0.223	0.108	0.833	+0.40 [−0.11, +0.89]
C (v7, v9-rerun, v10-rerun)	Clinical + human MRI	0.203	0.188	0.816	+0.46 [−0.04, +0.94]
D (v12, v16-rerun, v17-rerun)	Clinical + AI MRI	0.227	0.092	0.775	+0.54 [+0.03, +1.02]
E (v14, v15-rerun × 2)	Clinical + AI MRI + human LN	0.204	0.184	0.833	+0.40 [−0.09, +0.88]

All calibration metrics are based on the arithmetic mean of the predicted probabilities over three runs. BSS = Brier skill score (vs naive model; higher is better); E/O = mean predicted probability/observed event rate (ideal = 1.0; <1 indicates underestimation).

### 3.8. Risk-Factor Analysis

The factors the model cited most often for strategy E—parametrial invasion (99%), tumor size/bulk (96%), nodal status (87%), tumor markers such as SCC-Ag/CA125 (87%) and FIGO stage (77%)—are recognized prognostic variables, though citation frequency for a given factor varied across the five strategies (Figure 6), so we do not treat this ranking as strategy-independent. Citation frequency differed between patients who ultimately relapsed and those who did not: age (+6 points), vaginal/uterine involvement (+6) and hematologic indices (+6, including markers such as the neutrophil-to-lymphocyte ratio [37]) showed the largest differences, being cited more often in patients who relapsed, with smaller increases for LVSI (+3) and parametrial invasion (+2). Chemotherapy detail (−27) and histology/differentiation grade (−17) were the two largest differences in the opposite direction, being cited more often in patients who did not relapse, with a smaller decrease for FIGO stage (−4). These differences describe what the model mentioned in its free-text reasoning, not an independently validated predictor effect, and we did not fit a multivariable model on 40 events. Among the 40 patients who relapsed, the model’s stated recurrence pattern favored distant metastasis in 85% and local recurrence in 15% (strategy E; classified from the free text by keyword and stated-preference matching, Supplementary Methods S5).

## 4. Discussion

Two findings stand out. First, an off-the-shelf MLLM turned a clinical record plus an MRI report into a prognostic estimate that reached an AUC of 0.790 without any training. Moreover, most of the benefit over the clinical baseline came from the imaging report rather than from finer treatment detail, although this specific comparison was significant on our primary bootstrap test but not confirmed by a DeLong sensitivity analysis after multiplicity correction. Second, and against expectation, no statistically significant difference in AUC was detected between the AI-generated report and the radiologist’s MRI report in this exploratory cohort (strategies C versus D); this finding does not establish equivalence or non-inferiority, and the same AI report was, on inspection, often discordant with the reference report in specific and clinically important ways. Repeating each prompt three times allowed us to summarize run-to-run scatter (AUC SD 0.002–0.017), which is rarely reported for LLM predictions.

The report audit is the cautionary half of the story. The model was dependable on standardized signal features but discordant with the reference report exactly where a report changes management. For parametrial invasion and junctional-zone disease, the AI report was discordant with the reference report in roughly three cases in five (zero score on the corresponding rubric item); without an independent expert re-read of the underlying images, we cannot say how much of this reflects a genuine AI error versus an ambiguous or under-specified reference report; therefore, we describe it as discordance rather than confirmed overcalling. Of the two, only parametrial invasion is itself a FIGO-staging criterion, but both could still prompt unnecessary escalation if acted on uncritically. This pattern is compatible with a language-prior or confirmation-bias effect, but the present data cannot distinguish that explanation from the loss of 3D context in the supplied slices [15,29]. The nodal failure is more prosaic—the images we supplied did not show the nodes—but nodal status is an established prognostic factor [10], and this is precisely the gap that the human lymph-node description in strategy E addresses (D to E, ΔAUC +0.027; Holm *p* = 0.391, not significant). Confident statements unsupported by the reference report are a recognized safety concern for medical foundation models [30].

Set beside the literature, 0.790 is respectable for a zero-shot method: CerviPro reached 0.81 internally but 0.66–0.70 externally [13]; an international multicentre radiomic signature reached a comparable external-validation concordance index of 0.65 [17]; and LLM prognostication in laryngeal and hypopharyngeal cancer sat near 0.73 [27]. The staircase we saw—clinical about 0.70, plus an MRI report +0.06–0.08, plus nodal information +0.03—matches the known weight of imaging in this disease; the further increment from nodal information was not statistically confirmed in our sample (D vs. E, ΔAUC and 95% CI reported in Table 5, Holm *p* = 0.391), which we report as a point estimate with its uncertainty rather than as evidence that the true effect is small.

Two practical points follow. The three-run design showed that repeated calls at a temperature of 0.7 produced an observable run-to-run spread in AUC (SD 0.002–0.017 across strategies; not itself a confidence interval), and that richer input (strategy A) was numerically more variable than a terse one (strategy B). Reporting this dispersion across repeated runs seems prudent, though three runs only sketch it. Furthermore, every strategy underestimated its probabilities on average (E/O 0.78–0.83), so absolute risks were roughly 8–11 points too low on this sample. Any recalibration (for example Platt scaling) would need to be fitted and evaluated on an independent dataset or a properly nested resampling scheme, not on the same 82 patients used here, before the numbers were used at the bedside [35,38]. Because the model’s output is an uncalibrated, time-unanchored score rather than a probability at a prespecified decision threshold, a decision-curve analysis is not yet interpretable for this pipeline; calibration and a clinically defined action threshold are both prerequisites for a future decision-curve or net-benefit analysis.

The limits are those of an exploratory single-center series with 82 patients, which leaves the subgroup and cross-validation analyses with wide confidence intervals. The model input included the chemotherapy cycles and overall treatment time actually completed rather than only what was planned before treatment, so the prediction reported here is best understood as a retrospective, treatment-complete risk assessment rather than a strictly pretreatment one. A genuinely pretreatment-only version of strategies A, B and C would need to be re-evaluated using only fields available before treatment starts. The binary outcome merges local and distant relapse, the image cropping put lymph nodes out of reach, there was no external cohort, and a few early-stage patients treated with CCRT for clinical reasons remain in the cohort.

The reports were graded primarily by a model rather than by radiologists. An independent 18-case validation against two radiologists (Section 2.5.3, Table 3) found moderate-to-good but imprecisely estimated reliability between the two radiologists themselves (ICC 0.743, 95% CI 0.299–0.918) and only poor agreement between Claude and their consensus (ICC 0.283, 95% CI 0.077–0.496). The 68.8% macro-average figure is thus best understood as specific to the Claude-as-judge standard, not as an independently confirmed quality score. The parametrial-invasion finding was at least consistent with this check in the 10 cases where it applied for both raters, though that falls short of confirming it across the full cohort (Supplementary Methods S6). Three runs per strategy only sketch the output distribution, and the underestimation of absolute risk we observed still needs external recalibration. Because the original prompt did not specify a time horizon, we report the output as a risk score rather than a calibrated absolute probability.

Gemini’s training data likely include the general oncology literature, guidelines and terminology, which is expected of any general-purpose model and is not itself test-set leakage. The more serious concern is potential cohort-specific leakage: this cohort’s own patient records, outcomes or original reports appearing in training data, or outcome/follow-up information being inadvertently included in the model’s input. Our input audit found no recurrence labels or follow-up-time fields among the saved model inputs, though this check covers saved inputs rather than the full historical request log; patient identifiers were not publicly available, and this cohort has not been previously published or deposited. The imaging-benefit comparisons (A/B vs. C/E) were significant on our primary bootstrap test after Holm correction but were not confirmed by a DeLong sensitivity analysis; therefore, that conclusion should be treated cautiously pending a larger or external sample.

The human report was read from the full examination and is a clinical reference rather than a strict imaging gold standard, and no blinded expert re-read or adjudication of the underlying imaging has yet been performed. Findings such as parametrial overcalling therefore describe report-to-report discordance rather than a confirmed false-positive rate, and the lymph-node failure reflects the restricted image field of view rather than proven inferiority to radiologists. The classification metrics, while now cross-validated, remain imprecise at this sample size. The AI-generated report’s prognostic signal, despite its rubric discordances, could reflect genuine imaging information conveyed even in a partially discordant report, the model exploiting free-text patterns correlated with risk independent of factual accuracy, or both; our current design cannot distinguish these. We also did not run an image-occlusion or other interpretability analysis. Because Module 2 consumes only text, such an analysis would need to occlude regions of the input image at the Module 1 report-generation step and track how both the resulting report and the downstream risk score change, comparing lesion occlusion against area-matched non-lesion occlusion under controlled randomness. We treat this as a concrete next step rather than a claim that our current two-stage, image-to-text-to-prediction design itself demonstrates or rules out faithful image-based reasoning. Multicenter external validation, nodal-level images, a larger or full-cohort independent expert image re-read, and a prospective pilot are the natural next steps.

## 5. Conclusions

Used without fine-tuning, a general-purpose multimodal model combined a clinical record with a pelvic-MRI report and estimated recurrence after definitive radiotherapy with an AUC of 0.790. Most of the benefit over the clinical baseline came from the imaging report, though this comparison was significant on our primary bootstrap test but not confirmed by a DeLong sensitivity analysis after correction for multiple comparisons; AUCs were numerically higher for distant metastasis than local recurrence (0.810 versus 0.757). The same model’s MRI reports, however, scored only 68.8% against a clinical rubric and showed systematic discordance with the clinical reference report, including apparent overcalling of parametrial invasion, junctional-zone discordance and the inability to assess nodes within the supplied field of view. Therefore, they cannot yet stand unchecked, and an independent radiologist re-read would be needed before any of these as can be treated as confirmed errors rather than report discordance. Reporting the run-to-run variability under verified-consistent inputs, Holm-corrected multiplicity testing alongside a DeLong sensitivity check, and the disconnect between word-overlap metrics and clinical accuracy, we offer this pipeline and its baseline numbers as a starting point for multimodal language models in gynecologic radiation oncology. External validation, a larger or full-cohort independent expert image re-read, and prospective confirmation are needed before any clinical use.

## Figures and Tables

**Figure 1:**
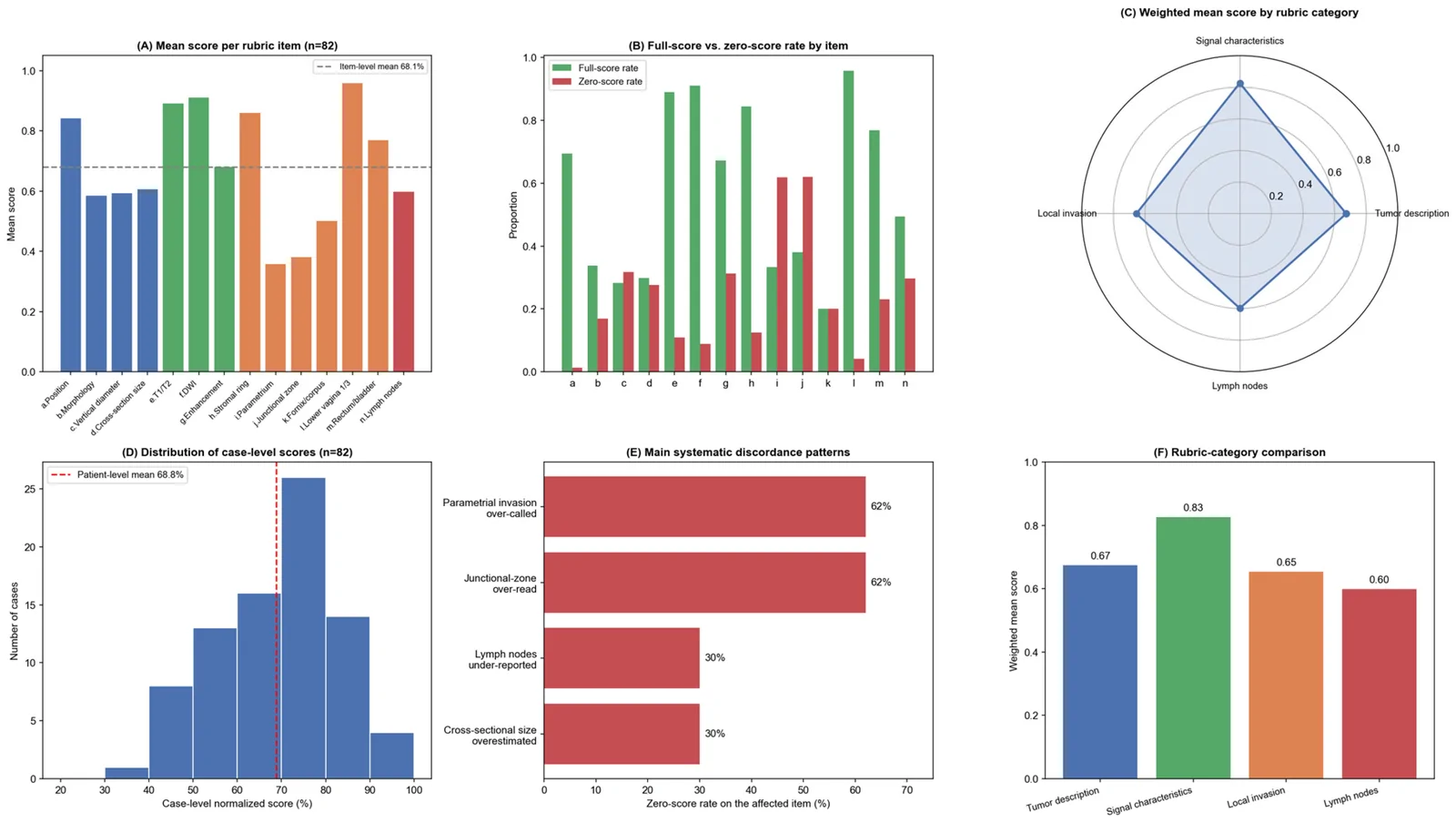
Rubric-based quality assessment of AI-generated MRI reports (n = 82). (**A**) Mean score of the 14 rubric items grouped by dimension, with the item-level macro-average (68.1%, the mean of the 14 item means) shown as a reference line—a different statistic from the patient-level mean in panel D; (**B**) full-score vs. zero-score rate per item; (**C**) radar plot of the weighted mean score of the four dimensions; (**D**) distribution of the case-level normalized rubric score (each case’s own item scores summed and divided by its own count of applicable items), with the patient-level mean (68.8%, Section 3.2) shown as a reference line; (**E**) the four systematic discordance patterns discussed in Section 3.2, expressed as the zero-score rate on the affected item; (**F**) the four dimensions from panel C compared directly.

**Figure 2:**
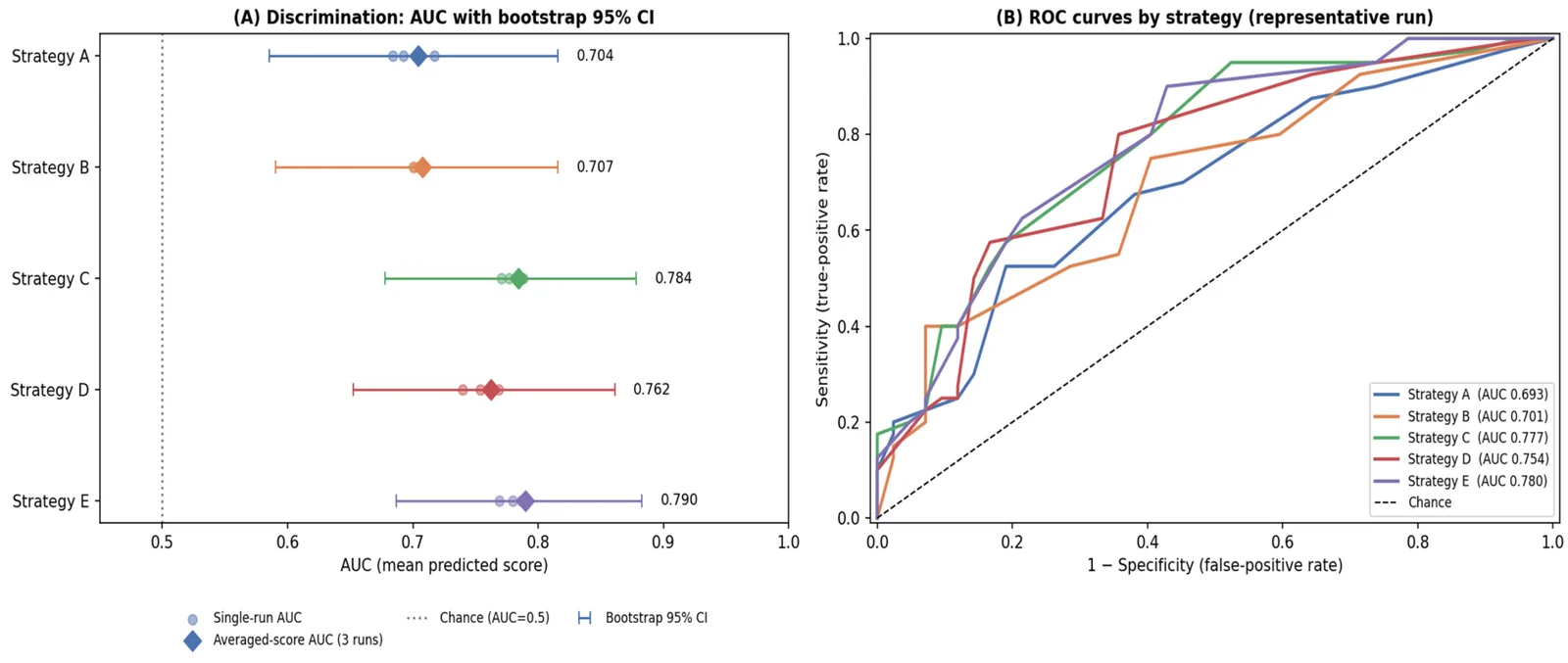
Discrimination of the prompting strategies (n = 82). (**A**) Mean-score AUC with bootstrap 95% confidence intervals; diamonds are the mean-score AUC for each strategy (averaged over three verified-input runs) and circles are the three individual-run AUCs. (**B**) ROC curves for a representative run of each strategy (the run whose single-run AUC was closest to the three-run mean); the AUC is given in the legend. Colors denote strategies consistently across both panels and across all figures.

**Figure 3:**
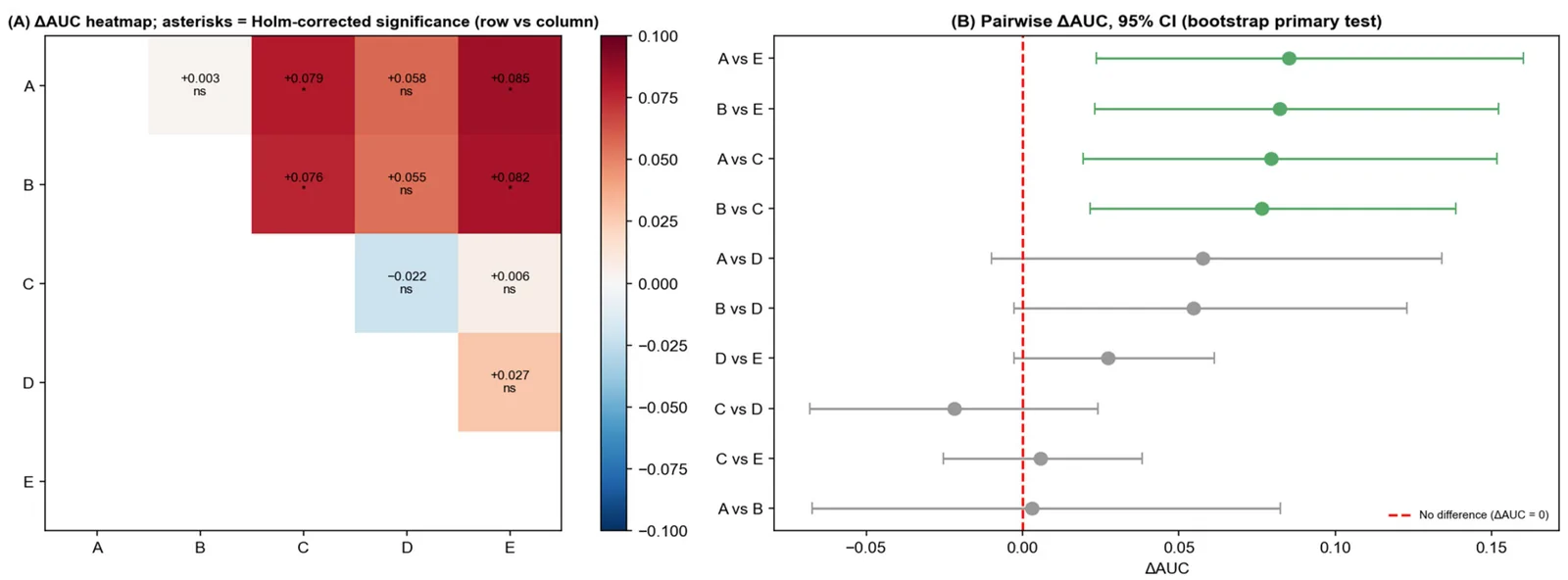
Pairwise comparison of strategies. (**A**) Heat-map of ΔAUC for all strategy pairs, with Holm-corrected bootstrap significance stars (row versus column); (**B**) key comparisons with ΔAUC and 95% bootstrap CIs (green = CI excludes zero, gray = CI includes zero; uncorrected). * *p* < 0.05; ns, *p* ≥ 0.05.

**Figure 4:**
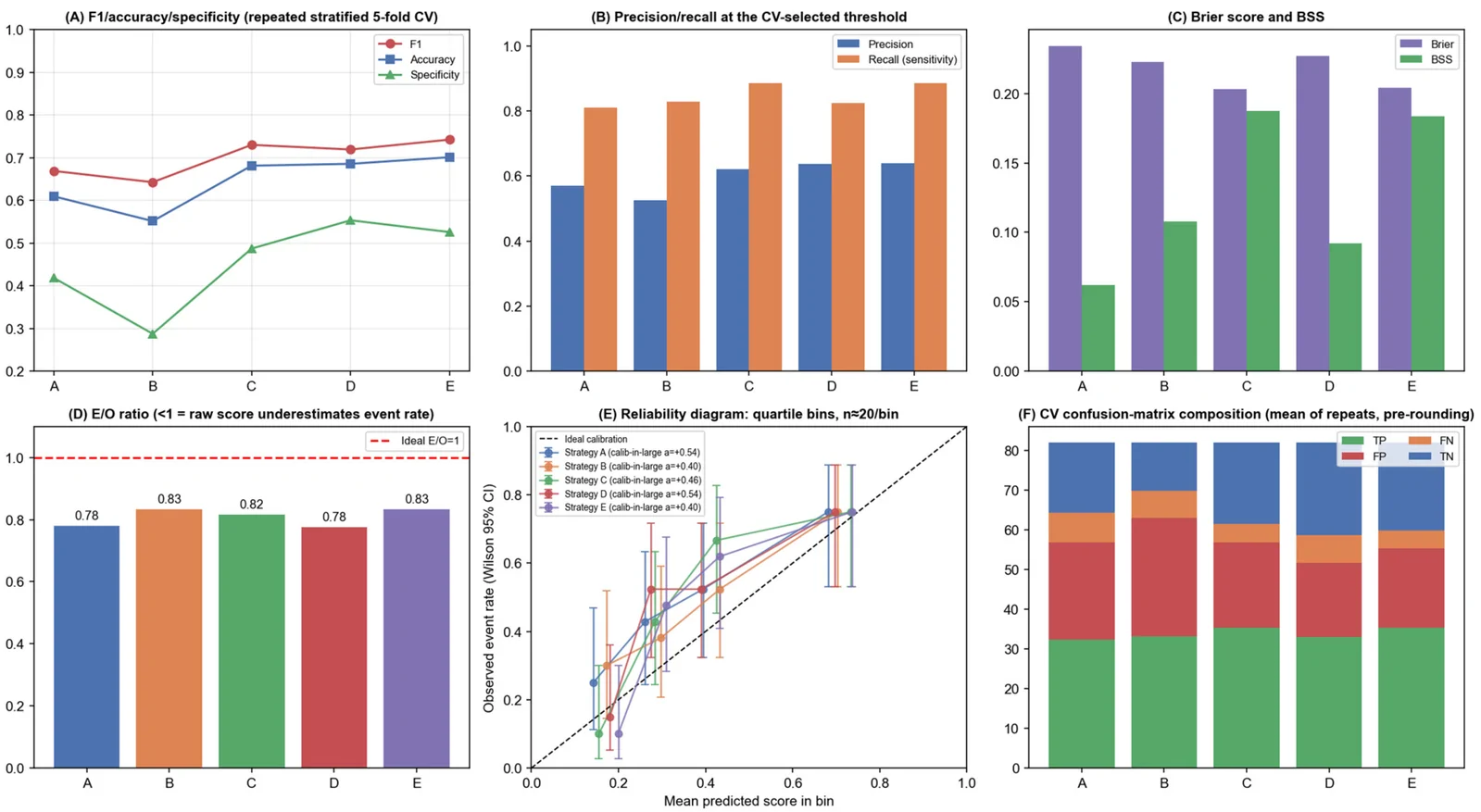
Classification performance and calibration (n = 82). (**A**) F1/accuracy/specificity from grouped, repeated stratified 5-fold cross-validation; (**B**) precision/recall at the cross-validated threshold; (**C**) Brier score and BSS; (**D**) E/O ratio; (**E**) reliability diagram: patients grouped into four quartile bins by predicted score, observed event rate per bin with Wilson 95% CI, plotted against the ideal-calibration diagonal; the calibration-in-the-large intercept (slope fixed at one) for each strategy is given in the legend as a summary reference, not as a substitute for the binned comparison; (**F**) cross-validated confusion-matrix composition (mean of CV repeats).

**Figure 5:**
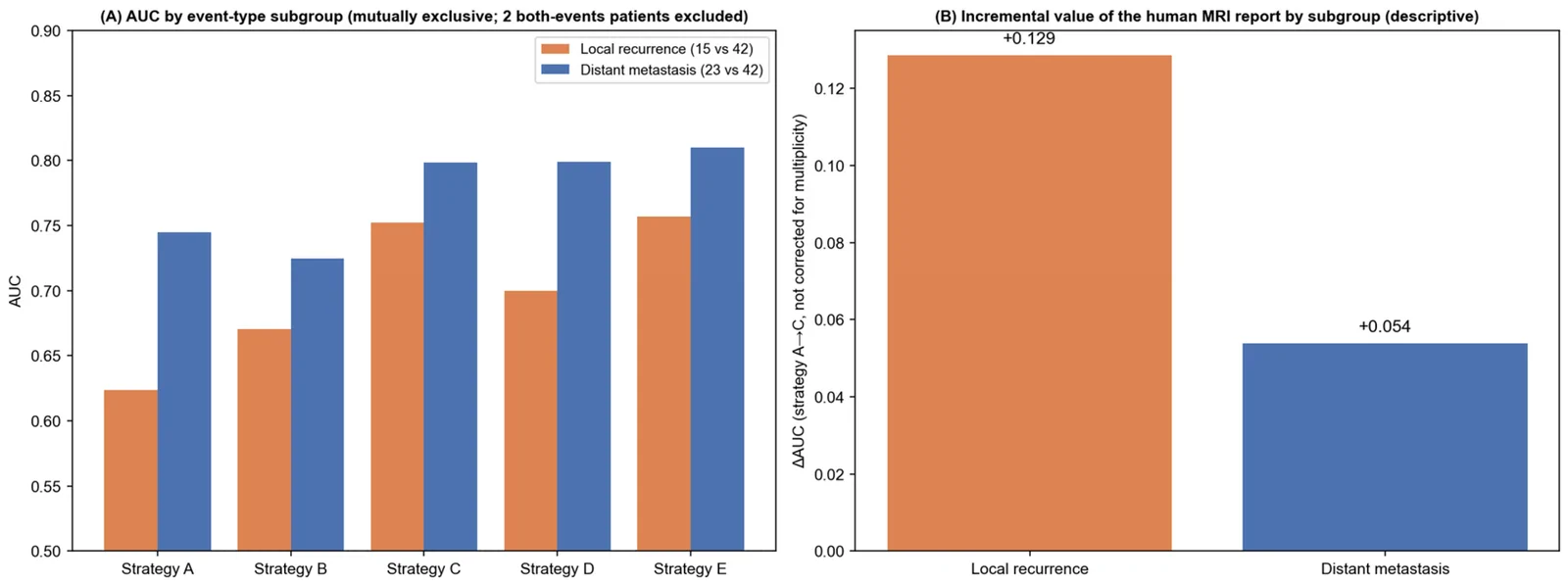
Event-type subgroup analysis (n = 82). (**A**) Subgroup AUC by strategy; (**B**) incremental value of MRI information (Strategy A → C) for each subgroup.

**Figure 6:**
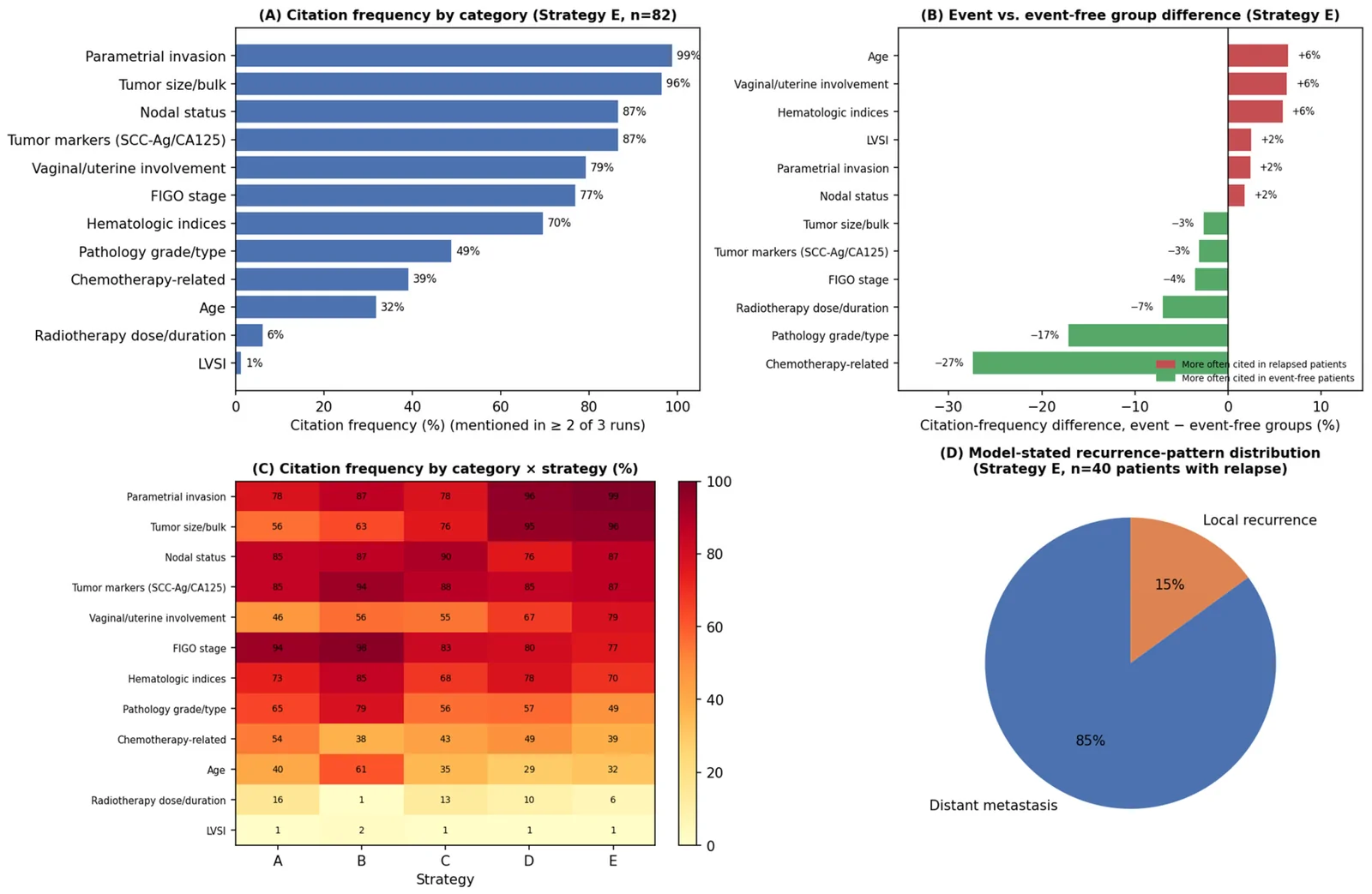
Risk-factor analysis (n = 82; recomputed directly from the free-text model outputs for this revision—see Supplementary Methods S5—rather than from the earlier illustrative summary). (**A**) Citation frequency by category for strategy E; (**B**) difference in citation frequency between patients who relapsed and those who did not; (**C**) citation frequency by category across all five strategies; (**D**) model-stated recurrence-pattern preference among the 40 patients who relapsed.

## Data Availability

The data presented in this study are available upon request from the corresponding author. The data are not publicly available owing to patient-privacy restrictions. The analysis code is available from the corresponding author upon reasonable request.

## Supplementary Materials

The following supporting information can be downloaded at: https://www.mdpi.com/article/10.3390/cancers18193136/s1, Figure S1, patient flow diagram; Table S1, case-level rubric scores for all 82 AI-generated MRI reports; Supplementary Methods S1–S4, the full prompt texts for Modules 1 and 2 (original Chinese and English translation) and the 14-item scoring rubric; Supplementary Methods S5, the risk-factor text-classification method; Supplementary Methods S6, the blinded expert report-review protocol; and Supplementary Code, the two scripts used to generate the AI MRI reports and the prognostic predictions analyzed in the manuscript (mri_report_generator_ENG.py, prognosis_analysis.py), with a redacted configuration template (config_template.py) in place of the authors’ own API key and file paths.

## Abbreviations

The following abbreviations are used in this manuscript:

Abbreviation	Definition
AUC	area under the receiver operating characteristic curve
BS/BSS	Brier score/Brier skill score
CCRT	concurrent chemoradiotherapy
CI	confidence interval
DWI	diffusion-weighted imaging
EBRT	external-beam radiotherapy
FIGO	International Federation of Gynecology and Obstetrics
IMRT	intensity-modulated radiotherapy
LLM	large language model
LN	lymph node
MLLM	multimodal large language model
MRI	magnetic resonance imaging
NLR	neutrophil-to-lymphocyte ratio
SCC-Ag	squamous cell carcinoma antigen
TOMO	helical tomotherapy
VLM	vision-language model
VMAT	volumetric-modulated arc therapy

## Appendix A

The complete case-level rubric scoring for all 82 AI-generated MRI reports (14 items per case, applicable-item count, total score and score rate; macro-average 68.8%) is provided as Supplementary Table S1.
