# Supplementary material for: Multimodal Large Language Models for Prognostic Prediction in Cervical Cancer Treated with Definitive Chemoradiotherapy: An Exploratory Study of Systematic Multimodal Data Integration
Source: Cancers (Basel). 2026 Sep 28;18(19):3136. doi: 10.3390/cancers18193136 (PMC13664468; doi:10.3390/cancers18193136)
Supplement: Supplementary file 1 [file cancers-18-03136-s001.zip › Supplementary_Methods_S1-S6.pdf]

Supplementary Materials

Multimodal Large Language Models for Prognostic Prediction in Cervical Cancer Treated with Definitive Chemoradiotherapy

This file contains the Supplementary Methods (prompt texts, prompting-strategy configurations and the report-quality rubric). Supplementary Figure S1 (patient flow diagram) and Supplementary Table S1 (case-level rubric scores for all 82 AI-generated MRI reports) are provided as separate files. The system prompts were originally written and executed in Chinese; faithful English translations are given below, with the operative instructions preserved.

Supplementary Figure S1. Patient flow diagram

Identification, eligibility screening, exclusions and the final analytic cohort, per the eligibility criteria in Section 2.1. Of 105 patients initially identified, 23 were excluded (13 without a complete pretreatment radiologist MRI report, 3 with incomplete treatment records and 7 with incomplete follow-up records), leaving the 82-patient analytic cohort used throughout this study.

Supplementary Methods S1. Module 1 — vision-language MRI report generation prompt (Gemini 3.1 Pro)

Role. You are a top-tier gynecologic-oncology radiologist with 20 years of experience. Your expertise is the precise assessment of tumor extent, local invasion and lymph-node status of cervical cancer from pelvic MRI. Your reporting style is objective, precise and highly concise, strictly serving clinical FIGO staging and balancing sensitivity (no missed findings) with specificity (no over-diagnosis).

Core reading principles and anti-error guidance (highest priority). Because only non-contiguous 2D static images are available, adhere to the following:

```
1. Precise measurement (avoid over-estimation and under-reporting): carefully distinguish solid tumor from edema, effusion or coexisting fibroids. Prefer 'lobulated' or 'irregular' for morphology. The maximum cranio-caudal (vertical) diameter MUST be assessed on the sagittal plane, and the axial cross-sectional size given with the scale bar.

2. Signal characteristics (mandatory): besides iso-T1 and mildly hyper-T2 signal, the report MUST include the DWI signal (usually hyperintense) and the enhancement pattern (cervical cancer typically shows relative hypo-enhancement compared with normal myometrium).

3. Local invasion (strict staging correspondence): (i) uterine corpus/fornix – assess whether the junctional zone is intact; report 'fornix involvement' or 'possible lower-corpus involvement' objectively if disrupted, but do not mistake intrauterine fluid for invasion; (ii) vagina – explicitly state, in standard staging terms, whether the lower one-third of the vagina is involved; (iii) parametrium – state whether the low-signal cervical stromal ring is continuous and whether strip-like infiltration is present.
```

```
4. Lymph-node assessment (objective, avoid false positives): describe small
   nodes objectively (e.g., 'multiple small nodes in the pelvic/bilateral inguinal
   regions') without prematurely concluding 'metastatic enlargement'; if none,
   report 'no significantly enlarged lymph node'. Do not hide genuinely present
   small nodes for fear of false positives.
```

Required four-part report structure. (1) Tumor location and morphology, including maximum vertical diameter and maximum cross-sectional size (mm); (2) signal characteristics (T1, T2, DWI) and a one-sentence summary of the enhancement pattern; (3) local invasion (parametrium; vagina/uterine corpus including junctional zone and lower vaginal third; rectum/bladder); (4) lymph nodes (pelvic and inguinal). The selected cropped slice from each sequence was supplied as a base64-encoded JPEG.

English translation.

You are a professional radiologist, skilled at interpreting MRI images and producing detailed medical reports.
Role
You are a top-tier gynecologic-oncology radiologist with 20 years of experience. Your specialty is precisely assessing cervical-cancer tumor extent, local invasion and lymph-node status from pelvic MRI images. Your reporting style is known for being "objective, precise and highly concise", strictly in service of clinical FIGO staging, holding a careful balance between "sensitivity (avoid missed findings)" and "specificity (avoid over-diagnosis)".

Core reading principles and anti-error guidance (highest priority)
Because you currently only see non-contiguous 2D static images, you must follow these principles:
1. Precise measurement (avoid both over-estimation and under-reporting): carefully distinguish the solid tumor from edema, effusion or a coexisting fibroid. Prefer "lobulated" or "irregular" for morphology. You must assess using the sagittal plane and report the "maximum vertical (craniocaudal) diameter", and give the axial cross-sectional size using the scale bar.
2. Mandatory signal features: besides iso-T1 and mildly hyper-T2 signal, you must include the DWI signal (usually hyperintense) and the enhancement pattern (cervical cancer typically shows "low enhancement" or a "reduced-enhancement region" relative to normal myometrium).
3. Local invasion assessment (rigorous correspondence to staging, do not miss subtle involvement):
   - Uterine corpus/fornix: focus on whether the junctional zone is clear. If the junctional zone is unclear or abnormal signal extends into the anterior/posterior fornix, objectively report "fornix involvement" or "possible lower-corpus involvement". Do not mistake simple endometrial-cavity fluid for invasion.
   - Vagina: you must use standard staging terminology to explicitly state whether the lower vaginal third is involved.
   - Parametrium: explicitly assess whether the low-signal cervical stromal ring is continuous, and whether there is strip-like infiltration.
4. Lymph-node assessment (objective, avoid false positives): if small nodules are seen, describe them objectively as "multiple small nodules/small lymph nodes in the pelvic/bilateral inguinal

regions” rather than readily concluding “metastatic enlargement”; if truly no abnormality is seen, report “no significantly enlarged lymph nodes”. Never withhold an objectively present small nodule out of fear of a false positive.

Report output structure
Output the report strictly under the following four dimensions, in highly concise language with no filler:
- Tumor location and morphology: specific location, morphology (e.g., lobulated/irregular), core signal (iso-T1, mildly hyper-T2, presence of long-T2 necrosis), must include the maximum vertical diameter and maximum cross-sectional size (mm).
- Signal characteristics: concisely describe T1, T2, DWI appearance, and must summarize the enhancement pattern in one sentence (e.g., low enhancement/reduced-enhancement region).
- Local invasion: parametrium (stromal ring continuity, parametrial invasion or not); vagina/uterine corpus (junctional-zone clarity, fornix and lower-corpus involvement; explicitly state whether the lower vaginal third is involved); rectum/bladder (fat-plane clarity, invasion or not).
- Lymph nodes: objectively describe pelvic and inguinal lymph-node findings (e.g., multiple small lymph nodes/nodules seen, or no significantly enlarged lymph nodes).

Now, based on the MRI images I have uploaded, please write the report following the structure and principles above.

Model and API metadata. Model: Gemini 3.1 Pro, called via the Poe API proxy (base_url = https://api.poe.com/v1) using an OpenAI-compatible chat-completions client. The production batch used for the MRI reports analysed here ran on 13 May 2026, 21:06:43–21:36:12 local time; the execution log records a 100% success rate. Temperature was not explicitly set. max_tokens, top_p and top_k were not set, so the provider defaults applied. No retry or back-off logic was implemented for this module; failed calls were logged and skipped. The per-image inputs were de-identified, cropped MRI slices as described in Section 2.3.

Supplementary Methods S2. Module 2 — prognostic-reasoning prompt (Gemini 3.1 Pro, text mode, temperature 0.7)

Base prompt. You are an experienced radiation-oncology specialist focusing on prognostic assessment of patients with locally advanced cervical cancer treated with definitive chemoradiotherapy. Based on the following patient information, first perform detailed analytical reasoning and then give a prognostic assessment. [Formatted patient data were then inserted.]

Analysis instructions (structured reasoning across six domains):

```
1. Clinical stage and tumor features – FIGO stage, tumor size, pathology; tumor volume/margins/signal on MRI; whether bulky (largest diameter >4 cm); location, extent and relation to adjacent organs; enhancement on T2 and T1+C.

2. Lymph-node status – nodal metastasis on imaging; size/location/laterality; short axis >1 cm regarded as positive.
```

```
3. Treatment technique – RT technique (IMRT/VMAT/TOMO); EBRT dose, nodal boost, brachytherapy dose; concurrent chemotherapy regimen and cycles; overall treatment time.

4. General status and haematology – age, performance status; haemoglobin; tumor markers (SCC-Ag, CA125); pretreatment lymphocytes, platelets, white cells, neutrophils.

5. Other clinical factors – general condition (e.g., age).

6. Integrated judgement – integrate favorable and unfavorable factors; estimate recurrence risk on an evidence-based basis.
```

Required output format (parsed automatically):

```
Recurrence/metastasis probability: [0–100]% (0 = certainly no recurrence/metastasis; 100 = certain; 50% = genuinely uncertain).

Main risk factors: [the 10 features from the input judged most influential for recurrence/metastasis].

Recurrence pattern: [distant metastasis (e.g., lung, supraclavicular nodes) vs in-field local recurrence (e.g., vagina, pelvis)].

Time to recurrence: [if recurrence is favored, the expected interval, e.g., 1, 3, 6 months, 5 years].
```

English translation.

You are an experienced radiation-oncology specialist, specializing in prognostic assessment of patients with locally advanced cervical cancer undergoing definitive chemoradiotherapy.
Based on the patient information below, first reason through a detailed analysis, then give a prognostic assessment.

Patient information: [every non-missing field for this patient is inserted here, including age, FIGO stage, hematology, treatment details, and the Module 1-generated MRI report text — see the field list in Sections 2.2–2.4]

Please follow this structure for a detailed analysis and assessment:

Detailed analytic logic

1. Clinical stage and tumor features: analyze FIGO stage, tumor size, pathology type, etc.; combine with image analysis of the MRI to assess tumor volume, margins and signal characteristics; assess whether the tumor volume is bulky (usually meaning a maximum diameter > 4 cm); analyze tumor location, extent of invasion and relationship to adjacent organs; pay particular attention to enhancement features on T2 and T1+C images.

2. Lymph-node status: assess nodal metastasis based on imaging; note node size, location and laterality; assess whether this is a high-risk factor (short axis > 1 cm is usually regarded as positive).

3. Treatment modality and technique: radiotherapy technique (IMRT/VMAT/TOMO, etc.); dose

assessment: external-beam dose, nodal boost, brachytherapy dose; chemotherapy regimen analysis: whether concurrent, drug type, number of cycles; treatment-time assessment: whether the overall treatment time was ideal.

4. General status and hematologic indices: general status such as age and performance status; the effect of hemoglobin level on radiotherapy outcome; the level and significance of tumor markers (such as SCC-Ag and CA125); the effect of other hematologic indices, e.g., pretreatment lymphocytes, platelets, white cells, neutrophils.

5. Other clinical factors: general patient status (e.g., age).

6. Integrated judgment: integrate all favorable and unfavorable factors; assess recurrence risk on an evidence-based basis; consider statistical data and clinical experience.

Assessment result (output strictly in the following format)

Recurrence or metastasis probability: [0–100]% (give a specific percentage; 0 means recurrence or metastasis is completely impossible, 100 means it is certain, 50% means genuinely uncertain about this patient's subsequent outcome)

Main risk factors: [extract the 10 features from the input patient data that you judge most influence recurrence or metastasis]

Recurrence pattern: [more inclined toward distant metastasis (e.g., lung, supraclavicular nodes) or in-field local recurrence (e.g., vagina, pelvis)]

Time to recurrence: [if the patient is judged likely to recur, indicate the expected interval, e.g., 1 month, 3 months, 6 months, 5 years]

Model and API metadata. Model: Gemini 3.1 Pro (Config.MODEL), called via the Poe API proxy (base_url = https://api.poe.com/v1) using a chat-completions-style request. The original production script did not log per-call timestamps; file-modification timestamps of the output files indicate that the batch was completed between 25 April and 1 June 2026. Temperature was 0.7. max_tokens, top_p and top_k were not set, so the provider defaults applied. Each call had up to three attempts, a 400-second request timeout, linear back-off after HTTP 429 responses (5 s × attempt number), and a 2-second pause before retrying other errors. The patient-information block was populated from non-missing fields as described in Sections 2.2–2.4.

Supplementary Methods S3. Prompting-strategy (ablation) configurations

Five strategies differed only in the input provided (an ablation of input modalities); each was run three times per patient (15 prediction sets in total).

- Strategy A — clinical + laboratory + full treatment information (EBRT technique, dose, brachytherapy scheme, chemotherapy cycles, etc.).

- Strategy B — clinical + laboratory + simplified treatment information (labelled only as 'definitive concurrent chemoradiotherapy').
- Strategy C — clinical + laboratory + treatment + human (radiologist) MRI report.
- Strategy D — clinical + laboratory + treatment + AI-generated MRI report.
- Strategy E – as strategy D, but with the AI-generated report's own lymph-node section text removed and replaced with a human lymph-node description (hybrid); this isolates the effect of replacing the AI's lymph-node text, not the effect of adding lymph-node information on top of an unchanged AI report.

Supplementary Methods S4. 14-item report-quality rubric (LLM-as-a-judge; Claude Opus 4.6)

Each AI report was scored against the paired human report on 14 items in four domains (1 = correct, 0.5 = partially correct, 0 = incorrect, N/A = not described in the reference and excluded from the denominator). Size items used a graded scale (error ≤5 mm = 1.0; ≤10 mm = 0.8; ≤15 mm = 0.5; >15 mm = 0).

Domain	Items
Tumor description	a Location; b Morphology; c Maximum vertical (cranio-caudal) diameter; d Maximum cross-sectional size
Signal characteristics	e T1/T2 signal; f DWI; g Enhancement pattern
Local invasion	h Stromal-ring continuity; i Parametrial invasion; j Junctional zone; k Fornix/uterine-corpus involvement; l Lower one-third of vagina; m Rectum/bladder
Lymph node	n Lymph-node assessment

The 82 report pairs were scored by an independent model from a different family (Claude Opus 4.6) so that judge and candidate shared no weights; each report was scored once (nine batches). Per-case scores are provided in Supplementary Table S1.

Supplementary Methods S5. Risk-factor text-classification method (Figure 6, Section 3.8)

Figure 6 and Section 3.8 are computed directly from the free-text "main risk factors" and "recurrence pattern" fields of the 15 underlying model-output files (three verified-input runs per strategy, A–E). A fixed set of clinical categories (parametrial invasion, nodal status, FIGO stage, tumor size/bulk, pathology grade/type, LVSI, tumor markers, chemotherapy-related, hematologic indices, vaginal/uterine involvement, radiotherapy dose/duration, age) was defined before extraction, using keyword matching against the risk-factor text; a patient-category pair was counted as "cited" if the keyword matched in at least 2 of the 3 runs for that strategy (majority

vote). Citation frequency per strategy = number of patients flagged / 82. The recurrence-pattern classification (local/distant) first looks for an explicit preference statement ("(more/slightly) leaning toward X") and classifies by that stated preference; only when no such statement is found does it fall back to keyword position. A file-mapping self-check (comparing each source file's predicted score against the official summary table, patient by patient) is run automatically before every use of this script; an internal review on 2026-09-17 found and corrected one file-mapping error (the third run of strategy E had been read from the wrong source file, matching only 40/82 patients against the official summary instead of 82/82), after which all 15 files were confirmed to match 82/82. The risk-factor categories and extraction rules are described here.

Supplementary Methods S6. Blinded expert report-review protocol and results

Objective. This validation asks whether the 14-item rubric scores that Claude Opus 4.6 assigned to AI-generated MRI reports (against the paired reference report) agree with the same scores assigned independently by human radiologists doing the same text-comparison task. It does not re-read the underlying MRI images, does not establish imaging ground truth for items such as parametrial invasion, and does not evaluate the prognostic-prediction modules.

Sampling. Eighteen of the 82 report pairs (about 22%) were drawn by stratified random sampling on outcome category (event-free, local recurrence, distant metastasis, both), with quota 9/3/5/1 respectively and a fixed random seed (20260916); this slightly exceeds the commonly used 10-20% range because the rare combined-event stratum (2 of 82 patients) was still given one case rather than dropped. The sample was fixed before scoring and not altered afterward.

Raters and blinding. Two senior attending radiologists each scored all 18 cases independently on 17 September 2026 under the fixed v2 protocol, using the same 14-item rubric and scoring scale as the original Claude Opus 4.6 evaluation (Supplementary Methods S4); the rubric and scale were fixed before scoring began and were not altered afterward. Case materials showed the reference (human-authored, full-examination) report and the AI-generated (cropped-image) report for each case; patient age and FIGO stage were omitted from the materials to avoid anchoring the parametrial and nodal items. Raters were blinded to Claude's prior item scores and rationale, to the patient's clinical outcome, and to the model's predicted risk score. Raters were not blinded to which report was the reference, since the scoring task is defined as AI-versus-reference comparison. Each rater scored independently, without access to the other rater's entries, before any discussion. The operational scoring rules (including the Applicable/N/A/Unclear classification criteria) were not refined or adjusted at any point between the original Claude Opus 4.6 evaluation and the two radiologists' scoring.

Scoring. Item scores were 0 (incorrect/discordant), 0.5 (partially correct) or 1 (correct/consistent), with items c and d (maximum craniocaudal diameter, maximum cross-sectional size) additionally allowing 0.8 for an error of >5-10 mm (size grading otherwise as in Supplementary Methods S4). Each item was first classified as Applicable, N/A (the reference report did not describe the item), or Unclear (the text was ambiguous and could not yet be classified under the operational rules;

raters recorded a reason rather than defaulting to N/A). Neither rater used the Unclear category in this sample.

Consensus and analysis plan. After both raters completed independent scoring, they discussed the items on which their scores differed (43 of 252 item-case combinations, spanning 16 of the 18 cases) and reached a consensus value for each; this consensus was used only as the reference for the Claude-versus-human comparison, never to recompute the raw inter-rater reliability estimate. Reliability was summarised as absolute-agreement ICC (two-way random effects, single measures) with patient-level bootstrap 95% CI (3,000 resamples), and, at the item level, linear-weighted Cohen's kappa (weight = 1 - |score difference|); we do not conflate this absolute-agreement ICC with a consistency-type ICC(3,1). Cases where an item's scores were tied across the sample (a near-ceiling item, e.g. lower-vaginal-third involvement) can produce an unstable or near-zero kappa despite small raw disagreement; we report both the kappa and the mean absolute difference for that reason.

Results. The two radiologists' independent scores agreed with each other at a level conventionally read as moderate-to-good (ICC 0.743, 95% CI 0.299-0.918; mean absolute difference 0.044 on the normalized case score), though the interval is wide at this sample size. Comparing Claude's scores to the human consensus formed after adjudication, agreement was conventionally read as poor (ICC 0.283, 95% CI 0.077-0.496; mean Claude score 0.648 versus 0.807 for the human consensus, i.e. Claude scored about 16 percentage points lower on average, consistently across nearly all 18 cases). Applicability judgments (Applicable vs. N/A) matched between Claude and the human consensus for 86.9% of the 252 item-case combinations; restricting the case-level score to only the items both judged applicable (a sensitivity analysis that removes applicability disagreement but also removes some of the comparison's scope) raised the Claude-versus-consensus ICC to 0.468, which we report alongside, not instead of, the primary 0.283 estimate. Item-level weighted kappa (Claude versus human consensus) was high for parametrial invasion (0.90, n=10 cases where both raters judged the item applicable) and for rectum/bladder (0.64); lower-vaginal-third involvement nominally reached 1.00, but this and other near-ceiling items (e, f above) are not directly comparable to 0.90, since a kappa computed on almost no raw disagreement is unstable rather than genuinely higher. Kappa was lowest for morphology (0.10), cross-sectional size (0.14), tumor location (0.27) and enhancement pattern (0.38). Full per-item results (n, exact agreement, mean absolute difference, weighted kappa for all three pairwise comparisons, and the applicability-matched sensitivity analysis) are provided in the data package (human_consensus_final.json and the accompanying item-level analysis file).

Interpretation. These results show that the LLM-as-a-judge rubric used for the full 82-case cohort is not interchangeable with independent human scoring on this text-comparison task, and is systematically stricter on several items; the 68.8% macro-average score (Section 3.2) should be read as specific to the Claude-as-judge standard. The parametrial-invasion item specifically was consistent between Claude and the human consensus on the 10 cases where it applied for both, which supports but does not independently confirm the parametrial over-calling finding discussed in the main text across the full cohort; no independent re-read of the underlying MRI images was performed, so this validation does not establish imaging ground truth.
